# Propylene–Ethylene Copolymer Covalent Adaptable Networks Synthesized by Resonance‐Stabilized, Radical‐Based Reactive Processing with Excellent Elevated‐Temperature Creep Resistance

**DOI:** 10.1002/cssc.202501137

**Published:** 2025-08-30

**Authors:** Yen‐Wen Huang, Mathew J. Suazo, Stephanie M. Barbon, Hayley A. Brown, Evelyn Auyeung, Colin Li Pi Shan, John M. Torkelson

**Affiliations:** ^1^ Department of Materials Science and Engineering Northwestern University Evanston IL 60208 USA; ^2^ The Dow Chemical Company Midland MI 48764 USA; ^3^ The Dow Chemical Company Lake Jackson TX 77566 USA; ^4^ Department of Chemical and Biological Engineering Northwestern University Evanston IL 60208 USA

**Keywords:** covalent adaptable networks, polyolefin, polypropylene, radical reactions, resonance stabilization, sustainable chemistry

## Abstract

Low‐crystallinity propylene–ethylene copolymer (PEC) thermoplastics exhibit creep in the melt and semicrystalline states. To enhance creep resistance while maintaining reprocessability, dynamic covalent cross‐links are introduced through one‐step, radical‐based reactive processing to create covalent adaptable networks (CANs). During reactive processing, it is essential to suppress *β*‐scission of propylene repeat units. To promote the formation of resonance‐stabilized macroradical intermediates, a methacrylate‐based cross‐linker bis(4‐methacryloyloxyphenyl) disulfide (BPMA) is replaced with a phenylacrylate‐based cross‐linker bis(4‐phenacryloyloxyphenyl) disulfide (BPST) and styrene and divinylbenzene, vinyl aromatic additives, are incorporated. The use of BPST but not BPMA leads to percolated PEC CAN formation. Adding vinyl aromatic additives reduces the disparity in cross‐linking capability between BPMA and BPST. The resulting PEC CANs show markedly improved elevated‐temperature creep resistance compared to neat PEC. Relative to thermoplastic PEC, the best‐performing PEC CAN suppresses >99% of viscous creep at 160 °C (melt state) over 600 s and >98% at 100 °C (semicrystalline state) over 10,000 s. This top‐performing PEC CAN is reprocessable through compression molding and twin‐screw extrusion, achieving full recovery of cross‐link density and tensile properties. These results showcase a promising one‐step strategy for producing recyclable PEC CANs with enhanced creep resistance in melt and semicrystalline states, addressing critical limitations of low‐crystallinity polyolefins.

## Graphical abstract

Propylene‐ethylene copolymer covalent adaptable networks (PEC CANs) are synthesized via one‐step, radical‐based reactive processing with the use of initiator, dynamic cross‐linkers, and vinyl aromatic additives. The best CAN suppresses over 98% of creep at 100 °C (semicrystalline state) for 10,000 s compared to neat PEC. The same CAN is reprocessable either through compression molding or twin‐screw extrusion.

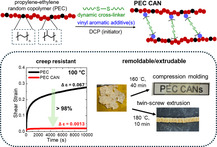

## 1 Introduction

Polypropylene (PP) is the second most produced plastic globally, accounting for ≈20% of worldwide plastic production.^[^ ^1^ ^]^ Most commercial PP is homopolymer, accounting for over 70% of the PP market, and the vast majority of PP homopolymer is isotactic PP (iPP).^[^ ^2^ ^]^ iPP is typically produced using conventional Ziegler‐Natta (Z‐N) catalyst systems, which promote stereospecific polymerization by selectively inserting propylene monomers in the same spatial orientation.^[^ ^3^ ^]^ iPP exhibits dense molecular packing, which is associated with its high crystallinity (>40%) and melting temperature (≈180 °C) as well as robust mechanical strength.^[^ ^4^ ^]^ These characteristics render iPP well‐suited for an array of applications including in medical devices and as automotive components and durable household goods.^[^ ^5^ ^]^ However, although the presence of extensive, rigid crystalline regions enhances strength, it also imparts some relative brittleness and limited impact resistance.^[^ ^6^, ^7^ ^]^ This restricts the utility of PP in applications where flexibility and resistance to sudden impact are essential.^[^ ^8^ ^]^

To address this limitation and bridge the performance gap between a rigid polyolefin and a soft elastomer, a low level of ethylene units, typically 1 to 30 mol%,^[^ ^9^, ^10^, ^11^ ^]^ may be incorporated during iPP polymerization, creating a propylene‐ethylene copolymer (PEC). Ethylene units disrupt the regularity of the PP backbone, reducing crystallinity and enhancing flexibility, elasticity, and optical clarity.^[^ ^9^, ^10^ ^]^ Precise control of monomer composition and sequence distribution allows tuning of these properties.^[^ ^11^ ^]^ The majority of propylene segments in PECs enhance compatibility with iPP, enabling improved processing, tunable mechanical properties, and better interfacial adhesion in blends. The type of catalyst used during copolymerization strongly influences the molecular structure and properties of the resulting PECs.^[^ ^11^ ^]^ Conventional Z‐N catalysts are classified as multisite catalysts, with each site exhibiting different reactivity toward comonomers.^[^ ^12^ ^]^ This variation, combined with the generally higher reactivity of Z‐N catalysts toward ethylene, often results in nonuniform, blocky copolymer sequences.^[^ ^13^ ^]^ Therefore, PECs made with Z‐N catalysts still exhibit significant semicrystalline characteristics, with crystallinity typically not falling below ≈15%.^[^ ^14^ ^]^ In contrast, single‐site catalysts such as metallocene or postmetallocene catalyst systems introduce greater randomness in the placement of ethylene units along the PP chain.^[^ ^14^ ^]^ This random incorporation further reduces the crystallinity of PEC, enabling the formation of thermoplastic elastomers.^[^ ^15^, ^16^ ^]^ In such systems, amorphous soft phases provide chain mobility for elastic deformation while the dispersed crystalline hard domains act as physical cross‐links to facilitate shape recovery.^[^ ^16^ ^]^ Low‐crystallinity PEC elastomers synthesized using advanced postmetallocene catalysts enable the production of materials with less than 5% crystallinity, yielding pronounced room‐temperature elastomeric behavior characterized by strain‐at‐break values exceeding 800% as well as excellent elastic recovery.^[^ ^16^ ^]^ PECs synthesized using metallocene or post‐metallocene catalysts exhibit a broad range of tunable properties, enabling their use across diverse applications, including as elastic films, gaskets, and sealants.^[^ ^17^ ^]^

Creep is the gradual, irreversible deformation under sustained load, which can undermine dimensional stability and mechanical performance over time.^[^ ^18^ ^]^ The high crystallinity of iPP strongly suppresses creep below its melting point.^[^ ^19^ ^]^ In contrast, low‐crystallinity PEC elastomers are susceptible to creep not only in the melt state but also in the semicrystalline state.^[^ ^20^, ^21^, ^22^ ^]^ To recover the creep resistance of PEC, postpolymerization cross‐linking can be introduced using peroxides in combination with cross‐linkers such as divinylbenzene (DVB)^[^ ^23^, ^24^ ^]^ or sulfur.^[^ ^25^ ^]^ Alternatively, vinyltrimethoxysilane^[^ ^26^, ^27^ ^]^ or maleic anhydride^[^ ^28^ ^]^ can be grafted onto the backbone of PEC and further reacted to form cross‐links. When percolated, permanent cross‐links are introduced by these methods, the resulting thermoset PEC networks cannot be recycled by melt‐state reprocessing, raising sustainability concerns.^[^ ^29^ ^]^

Dynamic covalent bonds offer a promising solution to overcome this issue.^[^ ^30^ ^]^ By introducing covalent bonds that are only reversible under certain stimuli (e.g., light, heat) as cross‐links, it is possible to form polymer networks with enhanced thermal and creep resistance as well as reprocessability.^[^ ^31^ ^]^ These networks, known as covalent adaptable networks (CANs), offer a promising route to reprocessable and high‐performance polyolefin elastomers.^[^ ^32^ ^]^ Two types of chemistry allow dynamic covalent cross‐links that can undergo reprocessing. First, dissociative dynamic chemistries, such as the Diels–Alder reaction,^[^ ^33^, ^34^ ^]^ alkoxyamine chemistry,^[^ ^35^, ^36^ ^]^ hindered urea exchange,^[^ ^37^, ^38^ ^]^ and dialkylamino disulfide chemistry,^[^ ^39^, ^40^ ^]^ proceed through reversible cleavage of covalent cross‐links, resulting in two reactive groups or radicals from a previously connected unit. Second, associative dynamic chemistries involve bond exchange processes where bond breaking and forming occur simultaneously; polymer networks with exclusively associative dynamic cross‐links are sometimes referred to as vitrimers.^[^ ^41^ ^]^ Associative‐type reactions include transesterification,^[^ ^42^, ^43^ ^]^ transamination,^[^ ^44^, ^45^ ^]^ boronic transesterification,^[^ ^46^, ^47^ ^]^ and siloxane exchange.^[^ ^48^ ^]^ Some dynamic covalent chemistries can proceed through both associative and dissociative pathways, such as those associated with aromatic disulfide,^[^ ^49^, ^50^ ^]^ urethane,^[^ ^51^, ^52^ ^]^ hydroxyurethane,^[^ ^53^, ^54^ ^]^ thiourethane,^[^ ^55^, ^56^ ^]^ and nonisocyanate thiourethane bonds.^[^ ^57^, ^58^ ^]^

**Figure 1:**
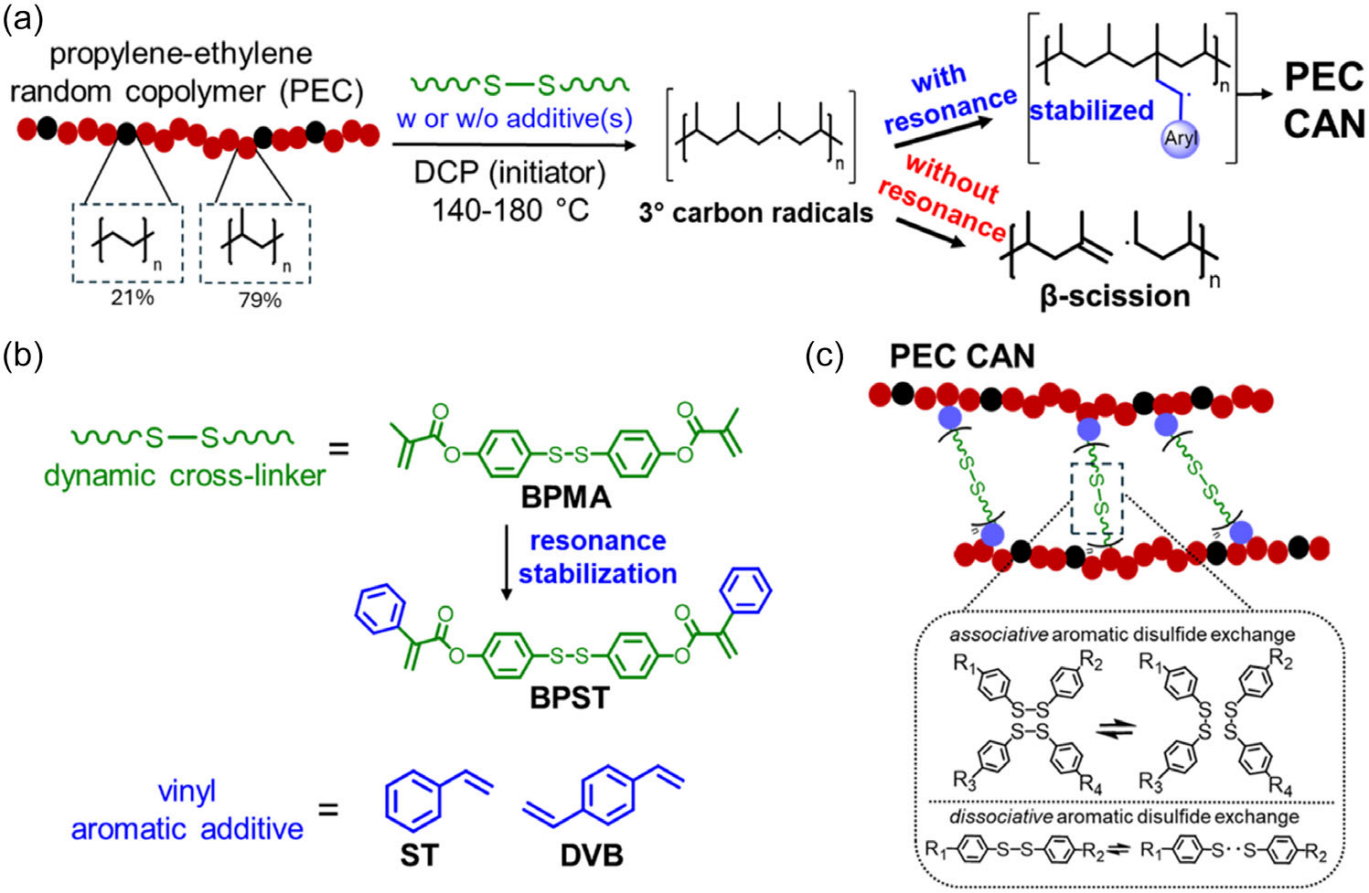
a) Synthesis of propylene‐ethylene random copolymer (PEC) CANs using a dynamic cross‐linker, vinyl aromatic additive(s), and an initiator. The initiator preferentially reacts with tertiary carbons in propylene units, where radicals tend to undergo β‐scission. Therefore, promoting the formation of a resonance‐stabilized intermediate from these tertiary carbon radicals is essential for PEC CAN formation. b) Resonance stabilization can be supplied by replacing BPMA (a methacrylate‐based dynamic cross‐linker) with BPST (a phenylacrylate‐based dynamic cross‐linker) and/or by adding vinyl aromatic additive(s) like ST and DVB. DVB is used in this work to introduce a low level of permanent cross‐links to enhance creep resistance. c) Structure and associative and dissociative aromatic disulfide dynamic chemistry of ideal PEC CANs.

Through post‐polymerization modification,^[^ ^59^ ^]^ dynamic cross‐links have been incorporated into both virgin and recycled polymers via a one‐step, radical‐based reactive processing approach.^[^ ^60^, ^61^, ^62^, ^63^, ^64^, ^65^, ^66^, ^67^, ^68^, ^69^, ^70^, ^71^, ^72^ ^]^ This approach has been successfully applied to polyolefins such as polyethylene,^[^ ^60^, ^61^, ^62^, ^63^, ^64^, ^65^, ^66^ ^]^ ethylene–octene copolymers,^[^ ^66^, ^67^ ^]^ olefin block copolymers,^[^ ^68^ ^]^ and most recently, ethylene‐vinyl acetate copolymer^[^ ^70^ ^]^ and PP.^[^ ^71^, ^72^ ^]^ As illustrated in **Figure** **1**a, during the cross‐linking of polyolefins, radicals generated from initiators (e.g., dicumyl peroxide, DCP) can abstract hydrogen atoms from polyolefin backbones, generating macroradicals.^[^ ^61^ ^]^ To form dynamic cross‐links, the macroradicals should graft onto both reactive sites of a bifunctional dynamic cross‐linker (such as those shown in Figure 1b) to form ideal polyolefin CANs (Figure 1c, and possible side reactions in Figure S1, Supporting Information).^[^ ^62^ ^]^ However, a previous attempt to introduce dynamic covalent bonds into a PEC using this one‐step approach was unsuccessful.^[^ ^67^ ^]^ As shown in Figure 1a, this limitation arose from the initiator's preferential reactivity toward tertiary carbons in the propylene units, with the resulting radicals predominantly undergoing *β*‐scission rather than participating in grafting or cross‐linking reactions.^[^ ^73^ ^]^

Consequently, most existing PEC CANs have been developed by incorporating a third monomer during copolymerization to introduce functional groups that enable dynamic cross‐linking.^[^ ^74^, ^75^, ^76^, ^77^, ^78^ ^]^ Examples of the third monomers include 8‐furyl‐1‐octene,^[^ ^74^, ^75^, ^76^ ^]^ 10‐undecen‐1‐ol,^[^ ^77^ ^]^ or boronic acid comonomers,^[^ ^78^ ^]^ which enable further cross‐linking with Diels–Alder,^[^ ^74^, ^75^ ^]^ imide bonds,^[^ ^76^ ^]^ disulfide bonds,^[^ ^77^ ^]^ or boroxine dynamic bonds.^[^ ^78^ ^]^ Reprocessability of PEC CANs was demonstrated in the studies by Wang et al.^[^ ^74^ ^]^ and Wu et al.^[^ ^77^ ^]^ based on Diels–Alder and disulfide bonds; however, the reprocessed samples recovered less than half of their original tensile strength after being damaged and undergoing repair.^[^ ^74^, ^77^ ^]^ Another PEC CAN study by He et al.^[^ ^76^ ^]^ was based on imide bond exchange and demonstrated full recovery of original mechanical properties within experimental uncertainty after reprocessing.^[^ ^76^ ^]^ PEC CANs made from unmodified PEC have only been demonstrated in a study by Ma et al. using a two‐step approach.^[^ ^79^ ^]^ In their study, benzophenone was first grafted onto PEC under UV light to introduce hydroxyl groups, which subsequently reacted to form networks that contained dynamic urethane, C—C, and hydrogen bonds.^[^ ^78^ ^]^ Although self‐healing and remolding of the cross‐linked PEC were demonstrated, recovery of mechanical properties was not evaluated.^[^ ^79^ ^]^ To the best of our knowledge, a direct, single‐step approach for forming reprocessable PEC CANs remains unexplored. Furthermore, creep suppression enabled by dynamic covalent bonds, which is particularly important for low‐crystallinity PEC elastomers, has not yet been investigated in any PEC CANs.

Recent advances in the development of PP CANs have addressed the challenge of *β*‐scission with vinyl aromatic additives during one‐step, radical‐based reactive processing.^[^ ^71^ ^]^ The strategy was inspired by earlier studies demonstrating that the addition of vinyl aromatic additives, such as styrene (ST),^[^ ^80^, ^81^ ^]^ DVB,^[^ ^82^, ^83^ ^]^ furan,^[^ ^84^ ^]^ thiophene,^[^ ^84^ ^]^ *p*‐(3‐butenyl)styrene,^[^ ^85^ ^]^ and 4‐vinylguaiacol,^[^ ^86^ ^]^ can suppress *β*‐scission and enhance grafting yield. These additives promote the formation of stabilized macroradical intermediates through resonance stabilization, thereby suppressing *β*‐scission pathways during radical‐based reactive processing (Figure 1a).^[^ ^86^ ^]^ The resulting PP CANs exhibit significant melt‐state creep suppression in comparison to neat PP and full cross‐link density recovery within experimental uncertainty after multiple reprocessing cycles.^[^ ^70^ ^]^ In a separate study,^[^ ^71^ ^]^ a styrene‐like moiety was incorporated into a methacrylate‐based dynamic cross‐linker (bis(4‐methacryloyloxyphenyl) disulfide; BPMA), yielding a phenylacrylate‐based cross‐linker (bis(4‐phenacryloyloxyphenyl) disulfide; BPST) (see Figure 1b). The presence of this styrene‐like structure imparts resonance stabilization, leading to improved cross‐linking capability compared to its methacrylate‐based analog.^[^ ^72^ ^]^ Notably, BPST enabled the formation of CANs even at low peroxide concentrations and with relatively low‐molecular‐weight PP, conditions under which BPMA was ineffective.^[^ ^72^ ^]^

Low‐crystallinity PEC elastomers require effective cross‐linking to suppress creep not only in the melt state but also in the semicrystalline state.^[^ ^87^ ^]^ Here, we aim to apply strategies that have proven effective in developing PP CANs to create highly creep‐resistant PEC CANs using a low‐crystallinity PEC elastomer.^[^ ^71^, ^72^ ^]^ Resonance stabilization is provided by replacing BPMA with BPST and/or by adding vinyl aromatic additives such as ST and DVB (Figure 1b). With a structure analogous to ST but containing a second double bond, a low level of permanent cross‐links introduced by DVB was shown to enhance creep resistance without sacrificing reprocessability.^[^ ^71^, ^88^ ^]^ In this work, we demonstrated that in the absence of vinyl aromatic additives, BPST, but not BPMA, can achieve percolated PEC networks after radical‐based reactive processing. Conversely, the use of one (ST) or two (ST and DVB) types of vinyl aromatic additives significantly minimizes the cross‐linking capability differences between BPMA and BPST by compensating for variations in resonance stabilization. Compared to neat PEC, the resulting PEC CANs exhibit superior creep suppression under various elevated temperature conditions. Significantly, compared to neat PEC, the best‐performing PEC CAN, formulated with BPST and a combination of ST and DVB, suppresses more than 99% of shear‐mode viscous creep at 160 °C (melt state) for 600 s and over 98% at 100 °C (semicrystalline state) as characterized over 10,000 s. Our best‐performing PEC CAN is reprocessable by compression molding or twin‐screw extrusion, resulting in full recovery of cross‐link density and tensile properties within experimental uncertainty. These findings suggest a promising one‐step strategy for developing reprocessable PEC CANs with improved creep resistance in both melt and semicrystalline states, addressing important limitations associated with low‐crystallinity thermoplastic polyolefins.

## 2 Results and Discussion

### 2.1 Synthesis and Characterization of Reactively Processed PECs

PEC CANs were synthesized via reactive processing using 1.0 wt% DCP and 0.10 mmol of dynamic cross‐linker per gram of PEC (either BPST or BPMA, corresponding to ≈4 wt%), with or without the addition of vinyl aromatic additives (ST or a combination of ST and DVB; detailed formulations are provided in **Table** **1**). Based on established procedures for peroxide‐induced cross‐linking of polyolefins,^[^ ^67^ ^]^ components were melt‐mixed at 140 °C for 3 min, followed by curing at 180 °C for 30 min in a compression molder to produce 1st mold samples. The FTIR spectra of neat PEC and reactive‐processed PEC are shown in Figure S4, Supporting Information. The naming convention of samples used throughout this study follows the format “PEC/cross‐linker/additive(s).” For example, PEC/BPMA/ST denotes a formulation containing DCP, BPMA, and ST.

**Table 1:** Formulation, thermal and thermomechanical properties, and gel contents of neat, thermoplastic PEC and 1st mold reactively processed PEC samples.

Sample	Additive(s) [mmol g PEC^−1^]	Gel contente)	*T*_m,peak_ f) [°C]	*T*_m,endpoint_ g) [°C]	χ_c_ h)	*E′* @160 °Ci)
Neat PECa)	0	0	142	154	4%	<0.01
PEC/BPMAa), b)	0	33 ± 5%	142	152	3%	0.023
PEC/BPSTa), b)	0	65 ± 3%	138	152	3%	0.16 ± 0.03
PEC/BPMA/STa), b)	0.20 (ST)c)	63 ± 5%	140	151	3%	0.31 ± 0.03
PEC/BPST/STa), b)	0.20 (ST)c)	74 ± 4%	139	151	3%	0.32 ± 0.03
PEC/BPMA/ST/DVBa), b)	0.20 (ST)c) + 0.05 (DVB)d)	77 ± 4%	137	150	2%	0.49 ± 0.04
PEC/BPST/ST/DVBa), b)	0.20 (ST)c) + 0.05 (DVB)d)	81 ± 6%	136	149	2%	0.52 ± 0.02

a) Precursor PEC contains 79 wt% of propylene units.

b) All samples (except neat PEC) include 0.10 mmol per g PEC of BPMA or BPST (≈4 wt%) and 1.0 wt% of DCP (calculated based on the mass of PEC in the mixer).

c) ≈2 wt% of ST (calculated based on the mass of PEC in the mixer).

d) ≈0.7 wt% of DVB (calculated based on the mass of PEC in the mixer).

e) From swelling tests with *o*‐xylene; error bars represent one standard deviation of three measurements.

f) Determined from DSC measurements of the melting peak, listed values are ± 1 °C.

g) Determined from DSC measurements of the end of the melting peak, listed values are ± 1 °C.

h) Crystallinity, calculated as the ratio of the latent heat of fusion from propylene units measured from DSC to the latent heat of fusion for 100% crystalline PP (207.1 J g^−1^),^[^ ^90^ ^]^ listed values are ± 1%.

i) Determined by DMA; error bars represent one standard deviation of three measurements.

**Figure 2:**
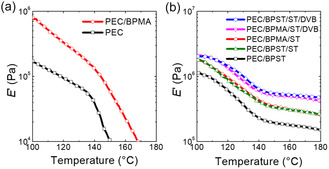
Tensile storage modulus (*E*′) as a function of temperature for neat PEC and the 1st mold of reactively processed PEC samples. The reactive processing of PEC was conducted at 180 °C using 1.0 wt% DCP and a) BPMA (0.10 mmol g^−1^ PEC); b) BPST (0.10 mmol g^−1^ PEC); BPST or BPMA (0.10 mmol g^−1^ PEC) with ST (0.20 mmol g^−1^ PEC); BPST or BPMA (0.10 mmol g^−1^ PEC) with ST (0.20 mmol g^−1^ PEC) and DVB (0.05 mmol g^−1^ PEC). The naming convention follows “PEC/cross‐linker type/additive type(s).” The addition of vinyl aromatic additives significantly minimizes cross‐linking efficiency differences between BPMA and BPST by compensating for variations in resonance stabilization.

The temperature‐dependent tensile storage modulus (*E'*) for neat PEC and the 1st mold of reactively processed PEC samples are presented in **Figure** **2**. Corresponding *E′* values at 160 °C are summarized in Table 1, and the tan δ profiles are provided in Figure S5, Supporting Information. When BPMA (a methacrylate‐based dynamic cross‐linker) was used, the resulting PEC/BPMA sample exhibited only a minority gel content (Table 1). No rubbery plateau was observed (Figure 2a), indicating a failure to form a cross‐linked network, and, therefore, that sample is not classified as a CAN. This result is consistent with our previous findings,^[^ ^66^ ^]^ which showed that methacrylate‐based cross‐linkers are ineffective for cross‐linking this specific PEC. In contrast, PEC/BPST, prepared using BPST, a phenylacrylate‐based dynamic cross‐linker containing additional phenyl groups that provide resonance stabilization, formed a network with a majority gel content of 65% and exhibited an *E′* value of 0.16 MPa at 160 °C (Table 1, Figure 2b). Table S1, Supporting Information, shows the maximum possible cross‐link density (86 mol m^−3^), calculated from the molar concentration of cross‐linker per unit volume of PEC. According to Flory's theory of ideal rubber elasticity, the modulus (*E*) in the rubbery plateau regime is linearly proportional to the effective cross‐link density (ν):^[^ ^89^ ^]^

(1) $$\begin{array}{c} \nu =E/\left(3RT\right) \end{array}$$

In the quasi‐rubbery plateau regime of our networks, the storage modulus *E′* (*T*) can be used to approximate *E*(*T*). Therefore, the experimentally determined cross‐link density for PEC/BPST is ≈15 mol m^−3^. This discrepancy from the maximum possible cross‐link density may be attributed to ineffective cross‐link loops and cross‐linker covalently attached on only one side, among other factors (Figure S1, Supporting Information), and suggests the potential for further enhancement.

Formulations containing 0.20 mmol ST per gram of PEC (≈2 wt%), the optimized concentration identified in a prior study,^[^ ^71^ ^]^ were also examined for both dynamic cross‐linkers, yielding two additional PEC CANs (PEC/BPMA/ST and PEC/BPST/ST). Both PEC/BPMA/ST and PEC/BPST/ST networks exhibited elevated *E′* values in the rubbery plateau compared to the corresponding formulations without ST, indicating increased cross‐link density (Figure 2b).^[^ ^89^ ^]^ Notably, the difference in cross‐linking capability between BPMA and BPST was substantially reduced, as evidenced by similar *E′* values at 160 °C within experimental uncertainty (≈0.3 MPa, see Table 1).^[^ ^89^ ^]^ These findings suggest that the inclusion of vinyl aromatic additives effectively compensates for variations in resonance stabilization, thereby minimizing the cross‐linking efficiency difference between BPMA‐based and BPST‐based networks.

The incorporation of a small amount of DVB has been shown to significantly enhance the creep resistance in PP CANs in the melt state while preserving reprocessability, owing to the introduction of a minor fraction of permanent cross‐links.^[^ ^71^ ^]^ Building on this strategy, we synthesized two additional PEC CANs using BPMA and BPST as dynamic cross‐linkers in combination with ST and DVB as vinyl aromatic additives. Specifically, DVB was added at 0.05 mmol per gram of PEC (≈0.7 wt%), alongside 0.20 mmol of ST per gram of PEC (≈2 wt%), consistent with the previously optimized formulation for PP.^[^ ^71^ ^]^ Both PEC/BPMA/ST/DVB and PEC/BPST/ST/DVB CANs exhibited further increases in cross‐link density (by approximately two‐thirds; see Figure 2b) and gel content (Table 1) relative to their ST‐only counterparts. As with earlier comparisons, no significant difference in cross‐link density was observed between these BPMA‐based and BPST‐based networks. To confirm that DVB cross‐links are below the percolation threshold, we treated the networks with dithiothreitol (DTT) to remove dynamic disulfide bonds.^[^ ^90^ ^]^ After the reactions, the residual gel contents were 7 ± 2% for PEC/BPMA/ST/DVB and 3 ± 2% for PEC/BPST/ST/DVB. These very low values indicate DVB‐derived permanent cross‐links are not effectively percolated. These findings reinforce the role of DVB in promoting the formation of highly cross‐linked PEC networks.

The thermal properties of reactively processed PECs were examined by differential scanning calorimetry (DSC) (Figure S6, Supporting Information), with melting transition peak (*T*_m,peak_), endpoint (*T*_m,endpoint_), and crystallinity (*χ*_c_) values listed in Table 1. Two distinct melting transitions were observed: a lower‐temperature endotherm (≈37–40 °C) attributed to imperfect crystallites formed during ambient annealing, and a higher‐temperature endotherm (≈140 °C) corresponding to stable propylene crystalline domains.^[^ ^16^ ^]^ Therefore, only the latter was used for crystallinity calculations. Both neat PEC and PEC CANs exhibited low *χ*_c_, ranging from 2 to 4%. Cross‐linking is known to reduce the lamella thickness of the crystal, leading to a further reduction in *χ*_c_, *T*_m,peak_, and *T*_m,endpoint_.^[^ ^91^, ^92^ ^]^ (In the case of PEC/BPMA, significant *β*‐scission also contributed to these reductions.^[^ ^91^, ^92^ ^]^) Consistent with this, the two PEC CANs with the highest cross‐link density (PEC/BPMA/ST/DVB and PEC/BPST/ST/DVB) exhibited the most significant reduction in *χ*_c_, *T*_m,peak_, and *T*_m,endpoint_ beyond experimental uncertainty compared to neat PEC.

Cross‐linking can also significantly influence the mechanical properties of low‐crystallinity PECs at room temperature. Representative stress–strain curves are shown in Figure S7, Supporting Information, and the corresponding values for Young's modulus, tensile strength, and elongation‐at‐break are summarized in **Table** **2**. Young's modulus and tensile strength are generally expected to decrease slightly due to the reduction in crystallinity caused by cross‐linking PEC into PEC CANs or by *β*‐scission.^[^ ^93^, ^94^ ^]^ However, in certain cases, the introduction of cross‐links effectively compensates for the loss of tensile properties associated with decreased crystallinity.^[^ ^93^, ^94^ ^]^ For instance, PEC/BPST/ST exhibited a tensile strength that exceeded that of neat PEC beyond experimental uncertainty. Similarly, PEC/BPMA/ST/DVB and PEC/BPST/ST/DVB showed improved tensile strength, with Young's modulus values recovering within experimental uncertainty compared to neat PEC.

**Table 2:** Room‐temperature tensile properties of neat PEC and 1st mold reactively processed PEC samples.

Sample	Young's modulus [MPa]a)	Tensile strength [MPa]a)	Elongation‐at‐break [%]a)
Neat PEC	13 ± 2	7.0 ± 1.4	950 ± 110
PEC/BPMA	9.0 ± 1.2	6.6 ± 1.5	1000 ± 100
PEC/BPST	7.0 ± 1.5	7.3 ± 1.0	1000 ± 100
PEC/BPMA/ST	7.1 ± 1.1	8.7 ± 1.0	1000 ± 100
PEC/BPST/ST	6.6 ± 1.4	10 ± 1	690 ± 70
PEC/BPMA/ST/DVB	15 ± 2	12 ± 1	330 ± 40
PEC/BPST/ST/DVB	16 ± 4	13 ± 2	330 ± 50

a) Obtained from tensile testing (strain rate = 0.38 s^−1^).

**Figure 3:**
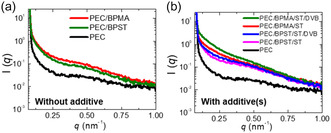
SAXS profiles of a) neat PEC and the 1st mold of reactively processed PEC samples without vinyl aromatic additives, and b) neat PEC and the 1st mold of reactively processed PEC samples with ST or a combination of ST and DVB as vinyl aromatic additives. Without vinyl aromatic additives, PEC/BPMA exhibits slightly higher scattering intensity, suggesting a slightly greater aggregation tendency of BPMA than BPST. With additives, BPMA‐based CANs show higher low‐q scattering intensity than BPST‐based CANs, indicating a more significant aggregation of BPMA in BPMA‐based CANs.

Phase separation during dynamic cross‐linking is also known to influence the mechanical performance of CANs.^[^ ^95^ ^]^ Previous studies suggest that microphase separation within nondynamic domains^[^ ^96^, ^97^, ^98^, ^99^, ^100^ ^]^ or within dynamic cross‐linker‐rich domains^[^ ^101^ ^]^ can restrict bond exchange and thereby suppress creep in CANs. However, in systems with both microphase and macrophase separation of dynamic cross‐linkers, such as polyethylene/dioxaborolane vitrimers, studies^[^ ^63^, ^64^ ^]^ suggest that these two types of phase separation exert opposite effects on viscoelastic behavior. The graft‐rich microdomains exhibit sluggish dynamics, whereas the cross‐link‐poor macrophase regions act as lubricants, facilitating flow during melt‐state processing.^[^ ^63^, ^64^ ^]^ In Figure S8a, Supporting Information, the neat PEC film appears optically transparent, while the PEC/BPST/ST/DVB film shows noticeable turbidity, suggesting the presence of macrophase separation in the PEC CAN sample. To investigate the microphase behavior of reactively processed PECs, small‐angle X‐ray scattering (SAXS) measurements were performed on 0.6 mm‐thick films (**Figure** **3**) at room temperature. Additionally, the SAXS profile for neat PEC at 160 °C is nearly identical to that obtained at room temperature (Figure S8b, Supporting Information), indicating that the observed features at room temperature are not attributable to crystalline lamellae.^[^ ^102^ ^]^ Furthermore, the increased SAXS intensity observed in PEC CANs compared to neat PEC cannot be solely ascribed to enhanced background contrast. The nonuniform intensity scaling across different *q* values (particularly in the low‐*q* region) and alterations in profile shape suggest that the incorporation of cross‐linker or additives induces nanoscale heterogeneities through partial microphase segregation.^[^ ^100^ ^]^ In the absence of vinyl aromatic additives (Figure 3a), PEC/BPMA exhibited slightly higher scattering intensity compared to PEC/BPST, suggesting a slightly greater tendency for aggregation. This observation is likely attributable to the enhanced solubility of BPST in the PEC matrix, which may result from its additional, less polar phenyl group. Aromatic hydrocarbons such as benzene, toluene, and xylene can swell or partially dissolve polyolefins at elevated temperatures.^[^ ^103^, ^104^ ^]^ Accordingly, low levels of ST and DVB are expected to be generally miscible with polyolefins under melt‐processing conditions. Notably, upon the addition of vinyl aromatic additives, BPMA‐based CANs exhibited a more pronounced increase in low‐*q* scattering intensity relative to BPST‐based CANs (Figure 3b). Less microphase separation of BPST‐based CANs is likely promoted by the phenyl group in BPST, which enhances compatibility with both the PEC matrix and the aromatic additives through favorable π–π stacking or van der Waals interactions.^[^ ^96^ ^]^ In contrast, cross‐links in BPMA‐based CANs may exhibit greater microphase separation from the matrix and additives.

### 2.2 Creep Resistance and Stress Relaxation of Reactively Processed PEC

**Figure 4:**
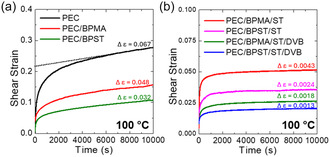
Creep curves for a) neat PEC and the 1st mold of reactively processed PEC samples without vinyl aromatic additives, and b) the 1st mold of reactively processed PEC samples with ST or a combination of ST and DVB as vinyl aromatic additives. Robust dynamic cross‐links in the reactively processed PEC samples enhance creep resistance at elevated temperatures. (As shown in (a) by the dashed line for the example of PEC, viscous creep strain (Δε) was calculated by extrapolating the slope of the best‐fit line from *t* = 9000 to 10,000 s back to *t* = 0 s and substracting the *y*‐intercept from the strain at t =  10,000 s.)

The elevated‐temperature creep resistance of PEC samples is strongly influenced by the presence and distribution of cross‐links. Creep behavior was evaluated under three conditions: “Set 1” with shear load of 3.0 kPa at 160 °C (melt state) for 600 s, “Set 2” with shear load of 3.0 kPa at 100 °C (semi‐crystalline state) for 10,000 s, and “Set 3” with tensile load of 0.65 MPa at 80 °C (semi‐crystalline state) for 24 h. For the “Set 1” and “Set 2” conditions, creep resistance was assessed using viscous creep strain (Δε). As shown in **Figure** **4**a, the value of this parameter is obtained by extrapolating the slope of the linear best‐fit line of the data between *t *= 500 s and *t* = 600 s (“Set 1”) or *t* = 9000 s and *t* = 10,000 s (“Set 2”) for the respective creep curve back to *t* = 0 s and subtracting this *y*‐intercept from the final strain value at *t* = 600 s or *t* = 10,000 s. The principle behind using Δε lies in isolating the time‐dependent viscous creep strain component (modeled by a dashpot in the Burgers model^[^ ^105^ ^]^). This metric provides practical relevance for long‐term loading applications and is especially useful for highly cross‐linked systems where terminal behavior is inaccessible within experimental timescales.^[^ ^106^ ^]^ For the “Set 3” condition, a direct comparison of accumulated strains was made.

Under Set 1 conditions, neat PEC and PEC/BPMA exhibited rapid flow, with viscous creep strains of 3600% and 1410%, respectively (Figure S9a, Supporting Information). In contrast, PEC/BPST, which contains a moderate cross‐link density, already demonstrated pronounced creep suppression (≈99% reduction) relative to neat PEC. The incorporation of vinyl aromatic additives further reduced creep in both BPMA‐ and BPST‐based systems (Figure S9b, Supporting Information). Notably, PEC/BPST/ST exhibited ≈40% lower viscous creep strain than PEC/BPMA/ST, despite similar cross‐link densities. Contrary to previous reports suggesting that microphase separation of dynamic cross‐linkers can enhance creep resistance,^[^ ^101^ ^]^ the more uniform cross‐link distribution observed in SAXS for BPST‐based networks appears to improve the creep resistance of CANs. Such uniformity likely enhances creep resistance in the melt state by preventing localized motion and promoting more even stress distribution.^[^ ^107^ ^]^ Samples containing a small fraction of permanent cross‐links (PEC/BPMA/ST/DVB and PEC/BPST/ST/DVB) showed even greater creep resistance, with roughly 70% lower viscous creep strain than their counterparts containing only ST. Consistently, PEC/BPST/ST/DVB outperformed PEC/BPMA/ST/DVB, exhibiting ≈30% lower viscous creep strain, again despite similar cross‐link densities. Power‐law exponents can be extracted from the slope of the creep compliance‐time plots on a log‐log scale between 500–600 s (Table S2, Supporting Information). The uncross‐linked PEC showed a slope of strain as a function of time near 1, consistent with terminal flow, while increasing cross‐link density reduced the slope to as low as 0.15, indicating increased elasticity.^[^ ^108^ ^]^ These results underscore the combined role of permanent cross‐links and uniform network structure in enhancing creep resistance under melt‐state conditions.

The enhanced creep resistance observed in the molten state was retained in the semicrystalline state. Under Set 2 conditions, creep suppression results (Figure 4) reflect the combined contributions of crystallinity and cross‐linking. Neat PEC exhibits the highest viscous creep strain (Δε = 0.067), while PEC/BPST shows moderately improved creep resistance (Δε = 0.032) due to the introduction of cross‐links. The incorporation of ST led to further improvements in creep resistance. Both PEC/BPMA/ST and PEC/BPST/ST exhibited substantial suppression of viscous creep strain, achieving ≈93–97% reductions compared to neat PEC. Samples containing both ST and DVB (PEC/BPMA/ST/DVB and PEC/BPST/ST/DVB) demonstrated the highest creep resistance, with PEC/BPST/ST/DVB exhibiting the lowest viscous creep strain (Δε = 0.0013), corresponding to a ≈98% reduction relative to neat PEC. These results highlight the synergistic effect of dynamic cross‐links and a small fraction of permanent cross‐links in suppressing creep deformation under semicrystalline conditions. Notably, when cross‐link densities were similar, BPST‐based CANs consistently outperformed their BPMA‐based counterparts in suppressing creep. To evaluate performance under more demanding conditions, neat PEC and PEC/BPST/ST/DVB were tested under Set 3 conditions, involving tensile loading (0.65 MPa) at elevated temperature (80 °C). After 24 h, PEC/BPST/ST/DVB exhibited ≈70% less creep than neat PEC (158% versus 50%, see Figure S10, Supporting Information). These results confirm that well‐formulated dynamic covalently cross‐linked networks can yield highly creep‐resistant PEC CANs.

Tensile stress relaxation measurements were performed on neat PEC and reactively processed PEC at 160 °C (Figure S11, Supporting Information). The stress relaxation data were fit using the Kohlrausch–Williams–Watts (KWW) stretched exponential decay function, and the resulting parameters were compiled in **Table** **3**. Noncross‐linked samples (PEC and PEC/BPMA) exhibited fast relaxation and could not be well fitted with the KWW function. In contrast, PEC CANs exhibited average relaxation times (<*τ*>) ranging from 28 to 168 s, generally aligning with the trend in cross‐link density. Consistent with the shear mode creep results at 160 °C, PEC/BPST/ST and PEC/BPST/ST/DVB exhibited slower stress relaxation compared to PEC/BPMA/ST and PEC/BPMA/ST/DVB, respectively, despite having similar cross‐link densities. These results confirm that the aromatic disulfide cross‐links effectively enable dynamic exchange behavior in these CANs.

**Table 3:** Characteristic relaxation times (τ*), stretching exponents (*β*) fitted from KWW function, and calculated average relaxation times (<*τ*>) for neat PEC, reactively processed PEC/BPMA, and 1st mold PEC CANs (PEC/BPST, PEC/BPMA/ST, PEC/BPST/ST, PEC/BPMA/ST/DVB, and PEC/BPST/ST/DVB) at 160 °C.

Sample	τ* [s]	β	<*τ*> [s]	R^2^
Neat PEC	3.9	1.00	3.9	0.428a)
PEC/BPMA	6.9	0.74	8.3	0.926a)
PEC/BPST	24	0.77	28	0.985
PEC/BPMA/ST	31	0.78	36	0.991
PEC/BPST/ST	39	0.69	50	0.991
PEC/BPMA/ST/DVB	62	0.49	129	0.981
PEC/BPST/ST/DVB	90	0.52	168	0.980

a) The low *R* ^2^ values indicate that the KWW function yields inadequate fits to noncross‐linked sample data.

### 2.3 Reprocessability of PEC CANs by Compression Molding and Twin‐Screw Extrusion

**Figure 5:**
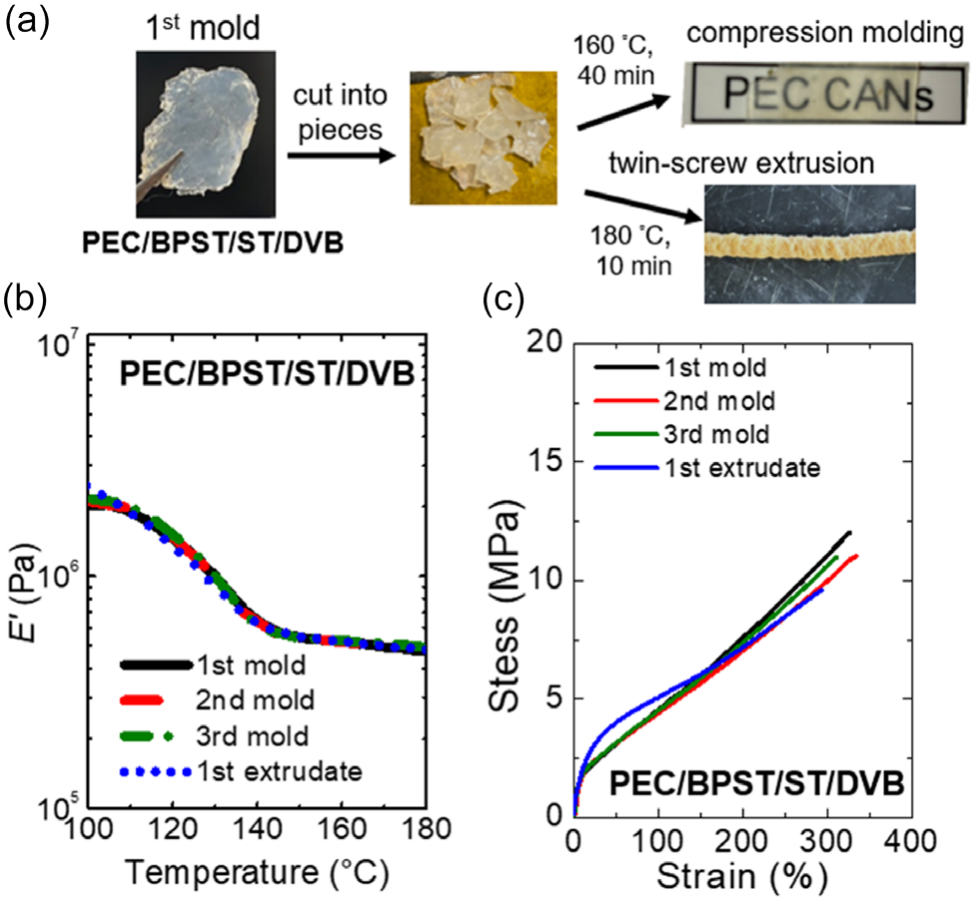
a) Image of 2nd mold and 1st extrudate reprocessed from fragmented pieces of 1st mold PEC/BPST/ST/DVB; b) tensile storage modulus (*E’*) and c) room‐temperature tensile stress–strain curves of 1st, 2nd, and 3rd mold samples as well as 1st extrudate of PEC/BPST/ST/DVB.

Reprocessability was first evaluated using PEC/BPST/ST/DVB, as it presented the greatest challenge due to its longest average stress relaxation time (168 s) and highest gel content among all samples. Reprocessing was carried out by either hot compression molding or twin‐screw extrusion. As shown in **Figure** **5**a, the 1st mold samples were fragmented into mm‐sized pieces before reprocessing. Compression molding at 160 °C for 40 min successfully consolidated the fragmented pieces into a continuous film (termed the 2nd mold sample). This process was repeated and successfully produced 3rd mold samples. Additionally, the fragmented 1st mold samples were fed into a HAAKE Minilab 3 extruder at 180 °C, yielding what is referred to as the 1st extrudate (Figure S12, Supporting Information). Shark‐skin defects observed in the 1st extrudate indicate flow instabilities during extrusion, typically caused by high shear stress at the die wall and discontinuities in flow at the die exit.^[^ ^109^ ^]^ These conditions lead to stick‐slip behavior of the extrudate, which manifests as surface irregularities.^[^ ^110^ ^]^ This phenomenon can be mitigated by modifying the die design, incorporating processing aids, or increasing the processing temperature to enhance flow and reduce stress accumulation.^[^ ^111^, ^112^, ^113^ ^]^ The temperature‐dependent *E′* profiles of the 1st, 2nd, and 3rd mold samples, as well as the 1st extrudate, are shown in Figure 5b (with *E′* values at 160 °C summarized in Table S3, Supporting Information). The rubbery plateau *E′* values across all reprocessed samples were consistent within experimental uncertainty, indicating full recovery of cross‐link density.^[^ ^114^ ^]^ Tensile properties (Figure 5c; Young's modulus, tensile strength, and elongation‐at‐break summarized in Table S4, Supporting Information) were also well maintained. Furthermore, DSC analyses (Figure S13, Supporting Information, with thermal properties summarized in Table S3, Supporting Information) confirmed the excellent retention of material properties after reprocessing.

The reprocessability of the other PEC CANs was similarly demonstrated using hot compression molding. Similar to PEC/BPST/ST/DVB, these samples also showed consistent recovery of cross‐link density and thermal properties following reprocessing (Figure S13 and S14, Table S3, Supporting Information). These results confirm that highly creep‐resistant PEC CANs can be produced via one‐step, radical‐based reactive processing. This approach may be extended in future work to investigate a range of PEC architectures, including random copolymers with varying comonomer sequence distributions or block copolymers, as well as copolymers with different ethylene contents. Moreover, the successful implementation of this method in PEC, a copolymer of ethylene and propylene, suggests its potential applicability to recycled plastic mixtures containing both polyethylene and polypropylene.^[^ ^115^, ^116^ ^]^

## 3 Conclusions

We presented a one‐step, radical‐based reactive processing strategy for synthesizing PEC CANs with significantly enhanced creep resistance. We demonstrated that resonance stabilization is critical for dynamic cross‐link formation: in the absence of vinyl aromatic additives, only the phenylacrylate‐based cross‐linker BPST (not the methacrylate‐based BPMA) successfully forms percolated PEC networks. However, the incorporation of ST or a combination of ST and DVB offsets differences in resonance stabilization, enabling both BPMA and BPST to form effective networks. The resulting PEC CANs exhibit exceptional suppression of viscous creep at elevated temperatures, with the best‐performing formulation (PEC/BPST/ST/DVB) achieving over 99% suppression at 160 °C and over 98% at 100 °C. Notably, at comparable cross‐link densities, BPST‐based CANs outperformed their BPMA‐based counterparts in creep resistance, which can be attributed to less phase separation and a more uniform distribution of cross‐links. We also demonstrated that the best‐performing CAN is fully reprocessable via both compression molding and twin‐screw extrusion, with complete recovery of cross‐link density and tensile properties within experimental uncertainty. Overall, these findings highlight a practical strategy to overcome the inherent creep limitations of low‐crystallinity polyolefins by introducing dynamic cross‐links through one‐step radical‐based reactive processing.

## 4 Experimental Section

4.1

4.1.1

##### Materials

Thionyl chloride (97%), 4‐(dimethylamino)pyridine (>99%), methacryloyl chloride (97%), dichloromethane (99.8%, anhydrous), N,N‐dimethylformamide (99.8%, anhydrous), potassium hydroxide (90%), triethylamine (99.5%), potassium carbonate (99%), magnesium sulfate (99.5%), chloroform‐d (CDCl_3_, 100%, 99.96 atom% D), dicumyl peroxide (DCP, 98%), *o*‐xylene (98%), ethanol (99.5%), dithiothreitol (DTT, 99%), styrene (ST, ≥99%), and divinylbenzene (DVB, 85%) were obtained from Sigma–Aldrich. 2‐Phenylacrylic acid (97%), acetonitrile (anhydrous, 99.8%), and chloroform (99.8%) were sourced from Fisher Scientific. Bis(4‐hydroxyphenyl) disulfide (98%) was purchased from Ambeed Inc. Neat PEC (VERSIFY 2400 Elastomer, 0.863 g cc^−1^, 2 MFR, estimated weight‐average molecular weight (M_w_) 400,000–1,000,000 g mol^−1^, with a dispersity (*Đ*) 2.0–2.3^[^ ^117^ ^]^), was received from The Dow Chemical Company. All chemicals were used as received without further purification.

##### Synthesis of BiPheS Methacrylate (BPMA) Dynamic Cross‐Linker

BPMA was prepared according to a previously reported method.^[^ ^49^ ^]^^1^H NMR (400 MHz, CDCl_3_): δ (ppm): 7.56–7.49 (m, 4H), 7.14–7.06 (m, 4H), 6.36 (t, 2H), 5.78 (p, 2H), 2.07 (t, 6H). Anal. calc'd. for [C_20_H_18_O_4_S_2_–NH_4_^+^]: 404.11; found: 404.52.

##### Synthesis of BiPheS α‐Phenylacrylate (BPST) Dynamic Cross‐Linker

BPST was prepared according to a previously reported method.^[^ ^71^ ^]^^1^H NMR (400 MHz, CDCl_3_): δ (ppm): 7.49 (t, 4H), 7.35 (d, 3H), 7.11 (d, 2H), 6.58 (s, 2H), 6.07 (s, 2H). Anal. calc'd. for [C_30_H_22_O_4_S_2_–NH_4_^+^]: 528.13; found: 528.13.

##### Characterization of Dynamic Cross‐Linkers

NMR spectra were acquired using a Bruker Avance III HD Nanobay 400 MHz spectrometer equipped with a SampleXpress autosampler, using CDCl_3_ as the solvent. Electrospray ionization mass spectrometry (ESI‐MS) was conducted on a Bruker AmaZon‐SL system, coupled with an Agilent 1100 Series HPLC, an ESI source, and a 3D ion trap analyzer. Samples were analyzed in positive ion mode using a mobile phase of 20% methanol and 80% dichloromethane. These characterizations of BPMA and BPST are provided in Figure S2 and S3, Supporting Information, respectively.

##### Reactive Processing of PEC

PEC pellets were combined with one of the dynamic cross‐linkers (BPMA or BPST), DCP, and optional additives such as ST and/or DVB in an Atlas Laboratory Mixing Molder, which was pre‐flushed twice with neat PEC to avoid contamination. To simulate the chaotic mixing typically observed in reactive extrusion, three steel balls were included in the mixer.^[^ ^90^ ^]^ The blend was made at 140 °C and 120 RPM for 3 min, with manual cycling of the rotor to enhance mixing. Afterward, the material was retrieved using a spatula and hot‐pressed into films (≈0.6 mm thick) using a PHI compression molding press (Model 0230C‐X1) at 180 °C under a 10‐ton ram force (≈8 MPa) for 30 min. These films were designated as the 1st mold PEC CAN samples.

##### Reprocessing of PEC CANs

The 1st mold PEC CANs were cut into mm‐sized fragments and reprocessed into 2nd mold samples by compression molding at 160 °C under a 10‐ton ram force (≈8 MPa) for 40 min using a PHI press (Model 0230C‐X1). The same procedure was repeated to convert the 2nd mold samples into 3rd mold samples. Alternatively, reprocessing was carried out via twin‐screw extrusion. A 1st mold PEC CAN (≈5 g) was chopped and fed into a HAAKE MiniLab 3 twin‐screw extruder (Thermo Scientific), operated at 180 °C and 40 RPM. The material was recirculated within the extruder for ≈10 min to ensure adequate mixing before being extruded. The extrudate was then cooled in air at room temperature to obtain the 1st extrudate samples.

##### Gel Content Determination

Neat PEC and PEC CANs (≈100 mg, *m*_0_) were immersed in *o*‐xylene at 140 °C, allowing the networks to swell significantly in the solvent. After 24 h, the liquid phase was removed, and the samples were then dried in a vacuum oven at 120 °C until their weight remained constant, providing the dried gel weight (*m*_d_). Gel content was calculated using the formula: gel content (%) = *m*_d_/*m*_0_ × 100%. For DVB‐containing PEC networks, a similar test was conducted, in which a 10‐fold molar excess of DTT relative to the disulfide bonds was added and reacted for 48 h at 140 °C before gel content was measured.^[^ ^90^ ^]^

##### Dynamic Mechanical Analysis

Dynamic mechanical analysis (DMA) in tension mode was performed using a TA Instruments RSA‐G2 Solids Analyzer on rectangular specimens. Tensile storage modulus (*E′*), loss modulus (*E″*), and damping ratio (tan δ, defined as *E″*/*E′*) were measured as a function of temperature from 100 to 180 °C. Experiments were conducted under airflow with a heating rate of 3 °C min^−1^, using a frequency of 1.0 Hz and an oscillatory strain amplitude of 0.03%.

##### Stress Relaxation

Stress relaxation measurements were carried out using a TA Instruments RSA‐G2 Solids Analyzer on rectangular specimens at 160 °C. A constant tensile strain of 5% was applied, and the relaxation modulus was recorded over time until it dropped below 15% of its initial value. The resulting data were analyzed using the Kohlrausch–Williams–Watts (KWW) stretched exponential function to characterize the relaxation behavior:^[^ ^118^ ^]^

(2) $$\begin{array}{c} \frac{E\left(t\right)}{E_{0}}=exp\left[{\left(-\frac{t}{\tau^{*}}\right)}^{\beta }\right] \end{array}$$

where *E*(t)/*E*_0_ represents the normalized relaxation modulus at time *t*, τ* denotes the characteristic relaxation time, and *β* (0 < *β* ≤ 1) is the stretching exponent that reflects the breadth of the relaxation time distribution. The average relaxation time, <*τ*>, is calculated as follows^[^ ^119^ ^]^

(3) $$\begin{array}{c} <\tau >=\frac{\tau^{*}\Gamma \;(\frac{1}{\beta })}{\beta } \end{array}$$

where Γ is the gamma function.

##### Differential Scanning Calorimetry

DSC was performed on ≈5 mg samples using a TA Instruments DSC 2500. Samples were sealed in hermetic aluminum pans and analyzed under a nitrogen atmosphere. Samples underwent two heating‐cooling cycles, each consisting of heating to 200 °C at a rate of 10 °C min^−1^, holding at 200 °C for 10 min, cooling to 0 °C at the same rate, and holding at 0 °C for an additional 10 min. The second heating cycle was used to determine the melting peak and endpoint temperatures from the endothermic melting transition. Crystallinity was calculated by integrating the higher temperature melting endotherm (≈140 °C) to obtain the enthalpy of fusion, which was then compared to the reference value of 207.1 J g^−1^ for 100% crystalline PP.^[^ ^114^ ^]^

##### Creep

Shear‐mode creep tests were performed by applying a constant shear stress of 3.0 kPa to disk‐shaped specimens (0.6 mm thick, 8 mm diameter) prepared from neat PEC and 1st mold films of reactively processed PEC. The experiments were conducted using an Anton Paar MCR 302e rheometer equipped with an oven‐hood and an 8‐mm parallel plate geometry. Prior to testing, samples were equilibrated at the test temperature for 5 min under a normal force of 1.0 N. The shear strain was measured over time under 3.0 kPa shear stress at 100 °C for 10,000 s or 160 °C for 600 s. Viscous creep strain (Δε, the gradual increase in shear strain due to viscous creep) was obtained by extrapolating the slope of the best‐fit line of the data between *t* = 9000 s and *t* = 10,000 s or between *t* = 500 s and *t *= 600 s for respective creep curve back to t = 0 s and subtracting this *y*‐intercept from the final strain value at *t* = 10,000 or *t* = 600 s.

##### Small‐Angle X‐Ray Scattering

Room‐temperature SAXS measurements were performed on the films with 0.6 mm thickness using a Rigaku S‐MAX 3000 instrument equipped with a Cu‐Kα source (*λ* = 0.154 nm) and calibrated with silver behenate. The sample‐to‐detector distance was set to 1640 mm. 2D scattering data were azimuthally averaged to generate 1D intensity profiles as a function of the scattering vector *q*, calculated by *q* = (4πsinθ)/λ, where θ is half the scattering angle. High‐temperature SAXS measurements were performed by cutting the films into thin strips and loading them into 2‐mm diameter capillary tubes, which are compatible with the instrument's high‐temperature sample stage.

##### Tensile Testing

Uniaxial tensile tests were performed at room temperature using an MTS Criterion testing system. Dogbone‐shaped specimens were prepared using a mold conforming to ASTM D1708 standards. A constant strain rate of 0.38 s^−1^ was applied during testing. Three specimens were tested, and standard deviations were reported as the error for Young's modulus, tensile strength, and elongation‐at‐break.

## Conflict of Interest

Some of the co‐authors are authors on a provisional US patent filing based on this research.

## Supporting information

Supplementary Material

## Data Availability

The data that support the findings of this study are available from the corresponding author upon reasonable request.
