# Supplementary material for: Propylene–Ethylene Copolymer Covalent Adaptable Networks Synthesized by Resonance‐Stabilized, Radical‐Based Reactive Processing with Excellent Elevated‐Temperature Creep Resistance
Source: ChemSusChem. 2025 Aug 30;18(20):e202501137. doi: 10.1002/cssc.202501137 (PMC12548940; doi:10.1002/cssc.202501137)
Supplement: Supplementary file 1 — Supplementary Material [file CSSC-18-e202501137-s001.pdf]

Supporting Information

Propylene–Ethylene Copolymer Covalent Adaptable Networks Synthesized by Resonance-Stabilized, Radical-Based Reactive Processing with Excellent Elevated-Temperature Creep Resistance

Yen-Wen Huang,[a] Mathew J. Suazo,[a] Stephanie M. Barbon,[b] Hayley Brown,[c] Evelyn Auyeung,[c] Colin Li Pi Shan,[c] and John M. Torkelson[a,d,*]

[a]Dept. of Materials Science and Engineering, Northwestern University, Evanston, IL 60208, United States

[b]The Dow Chemical Company, Midland, MI 48764, United States

[c]The Dow Chemical Company, Lake Jackson, TX 77566, United States

[d]Dept. of Chemical and Biological Engineering, Northwestern University, Evanston, IL 60208, United States

[*]Corresponding author: j-torkelson@northwestern.edu

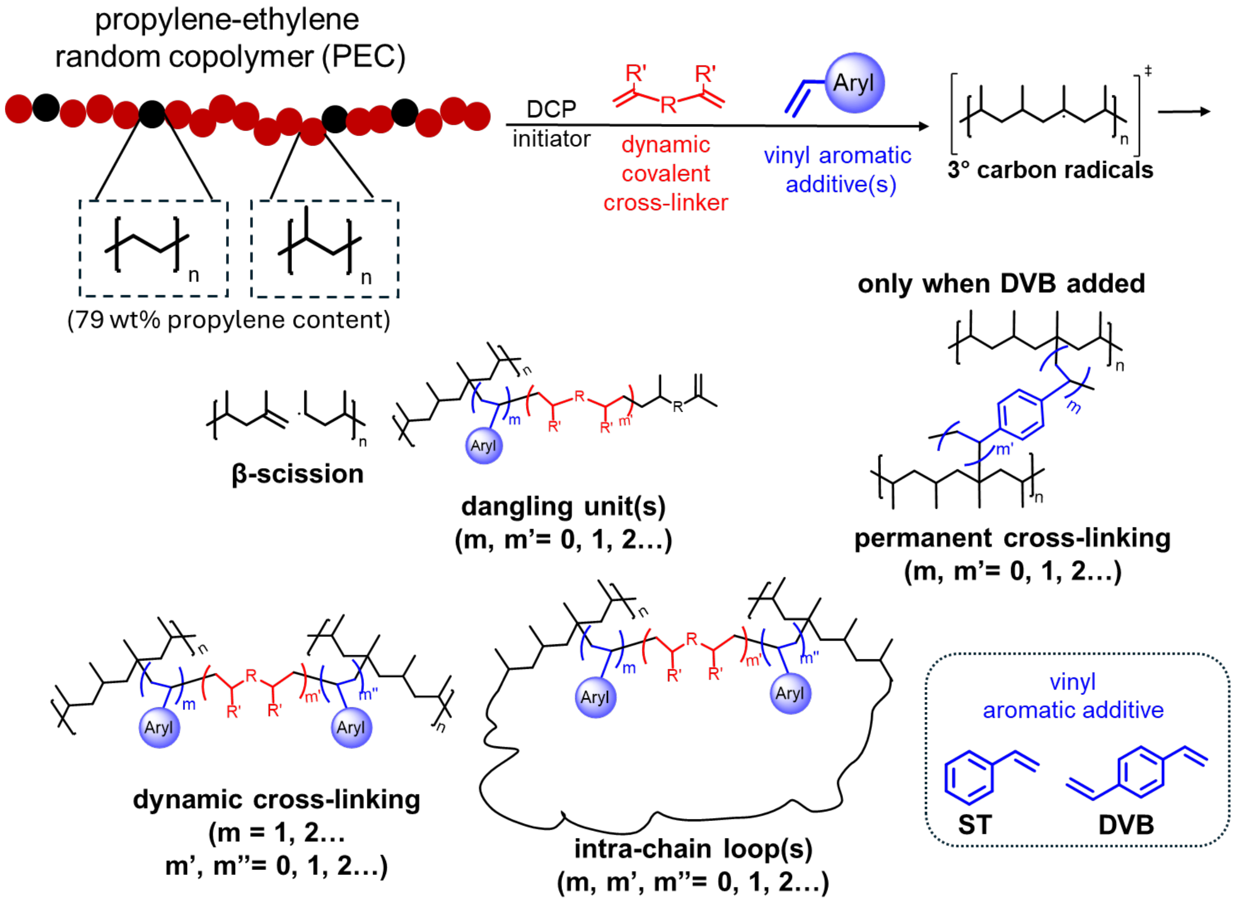

Figure S1. Possible reactions during radical-based reactive processing of PEC copolymer with dynamic covalent cross-linkers, initiator, and vinyl aromatic additives at 180 °C. Permanent cross-links happen only when divinyl benzene (DVB) is used as a vinyl aromatic additive. Adapted with permission from *Macromolecules* **2025**, 58, 9, 4847–4859. Copyright 2025 American Chemical Society.

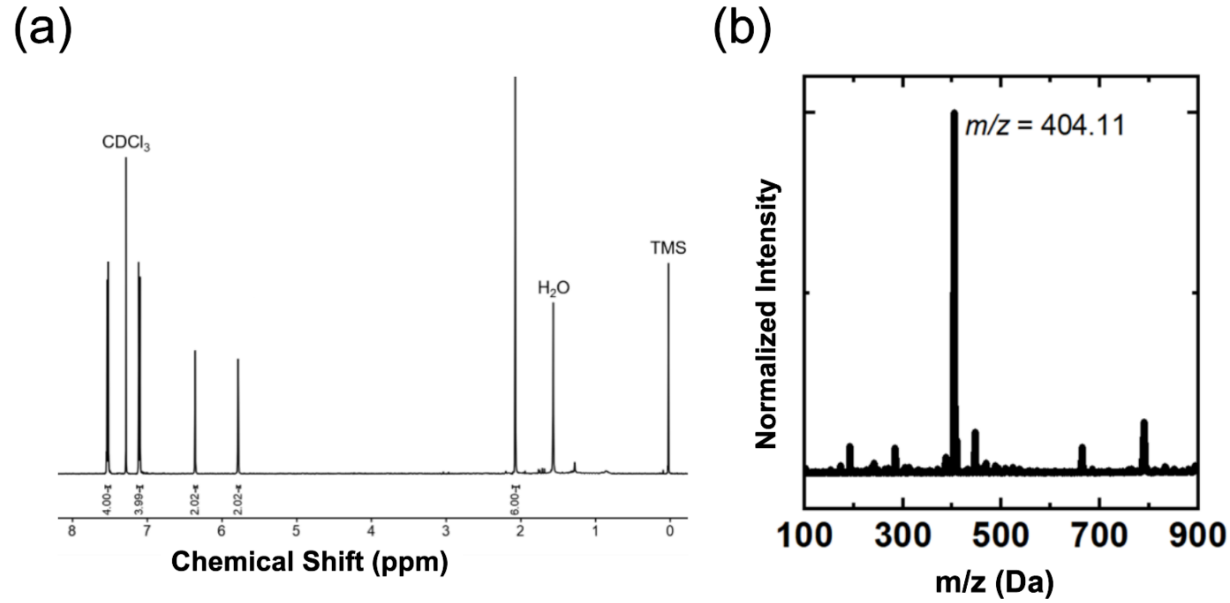

Figure S2. (a) ^{1}H NMR spectrum and (b) ESI-MS spectrum of BPMA.[S1]

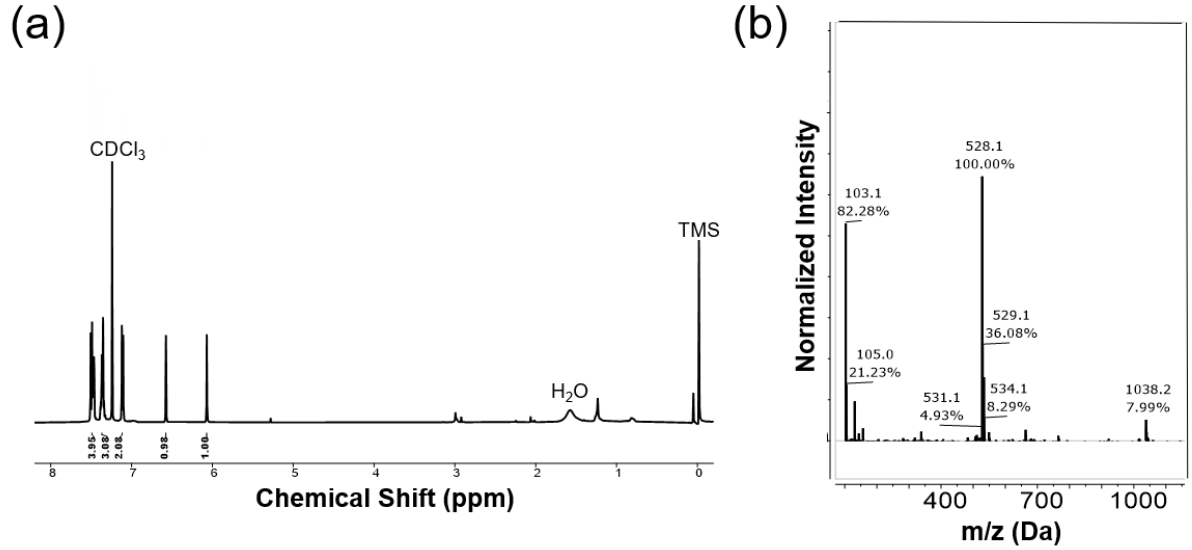

Figure S3. (a) ^{1}H NMR spectrum and (b) ESI-MS spectrum of BPST.[S2]

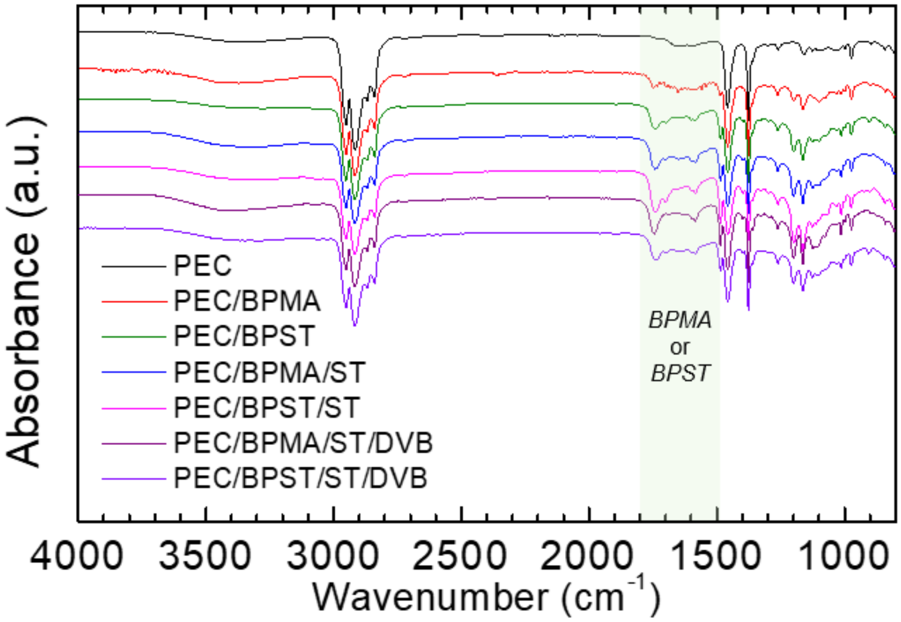

Figure S4. FTIR spectra of neat PEC and reactively processed PEC.

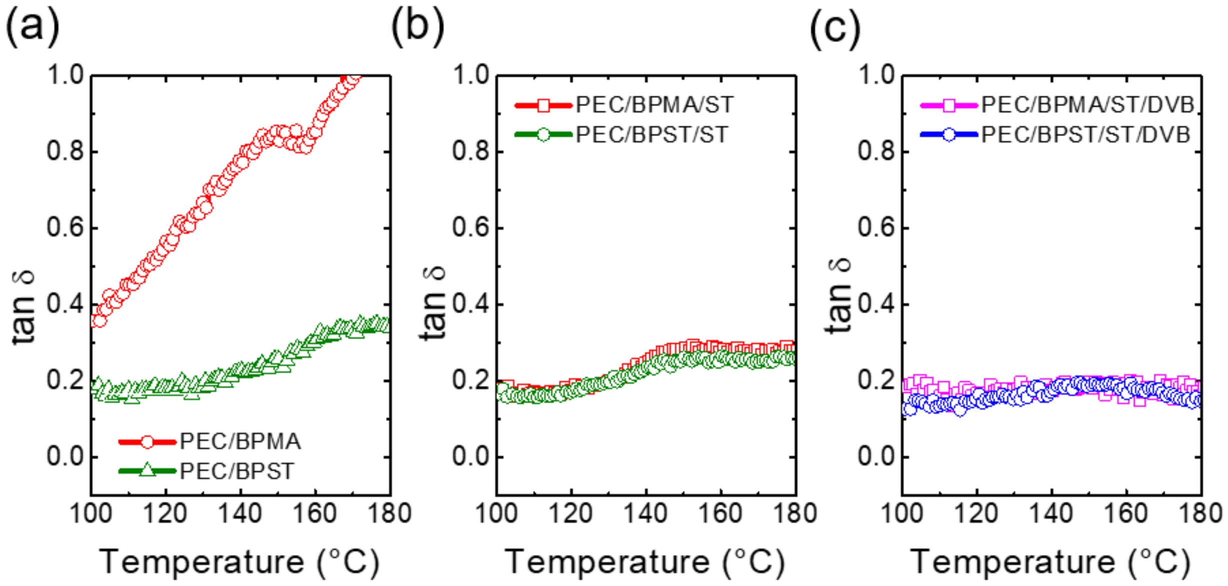

Figure S5. Tan δ (*E*''/*E*') as a function of temperature. These samples were synthesized at 180°C using 1.0 wt% DCP and (a) BPMA or BPST (0.10 mmol/g PEC), (b) BPST or BPMA (0.10 mmol/g PEC) with styrene (ST, 0.20 mmol/g PEC), and (c) BPST or BPMA (0.10 mmol/g PEC) with ST (0.20 mmol/g PEC) and DVB (0.05 mmol/g PEC).

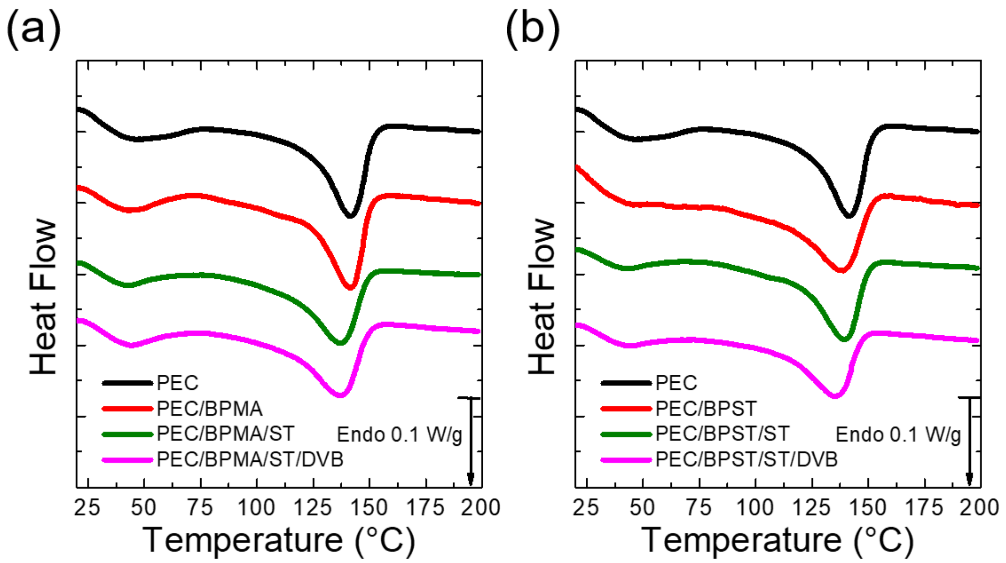

Figure S6. DSC thermograms for neat PEC and 1st molds of reactively processed PEC samples using (a) BPMA or (b) BPST.

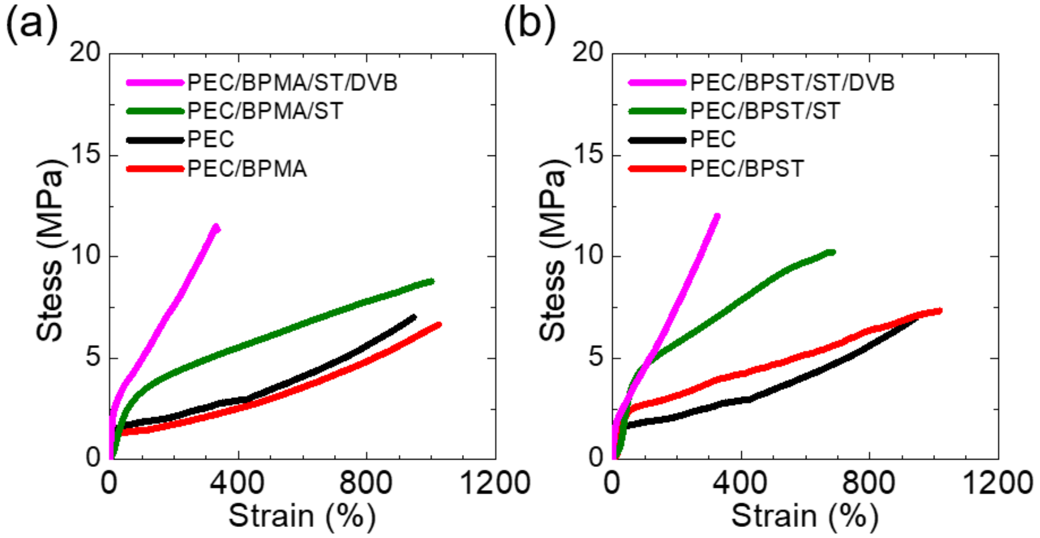

Figure S7. Room-temperature stress-strain curves of neat PEC and 1st molds of reactively processed PEC samples using (a) BPMA or (b) BPST.

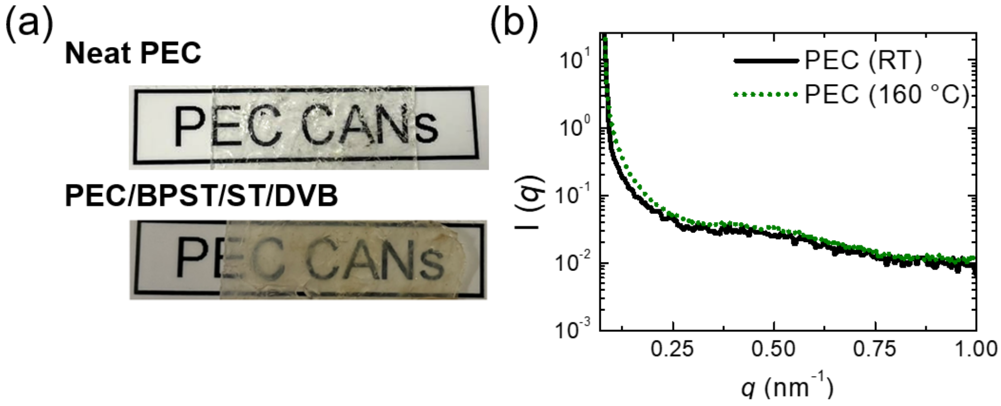

Figure S8. (a) Photographs of 1st mold neat PEC and PEC/BPST/ST/DVB films: the neat PEC film appears optically transparent; however, the PEC/BPST/ST/DVB film shows noticeable turbidity, suggesting the presence of macrophase separation in the PEC CAN sample. (b) SAXS profiles of neat PEC at room temperature and 160 °C, indicating the lack of structure factor contributed by the crystal lamellae at room temperature.

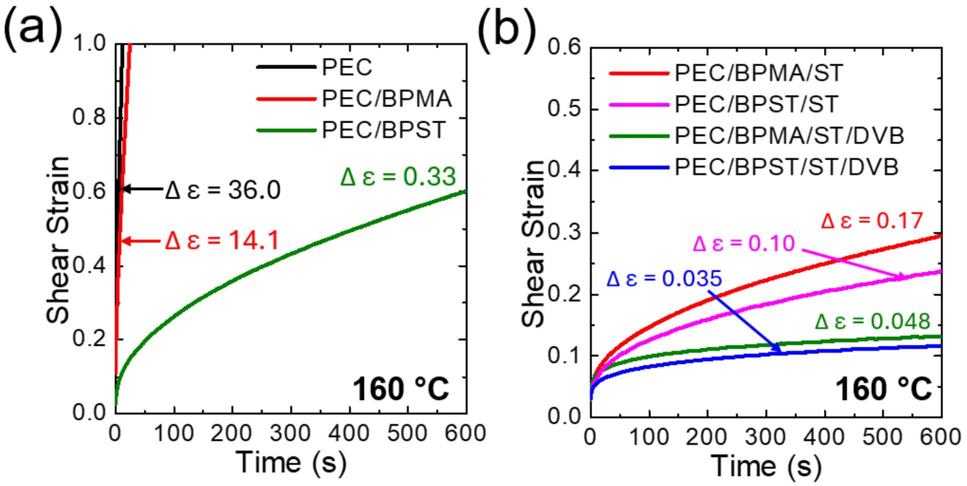

Figure S9. Creep curves for (a) neat PEC and the 1st mold of reactively processed PEC samples without vinyl aromatic additives, and (b) the 1st mold of reactively processed PEC samples with ST or a combination of ST and DVB as vinyl aromatic additives. The shear strain was measured over time under 3.0 kPa shear stress at 160°C for 600 s. Viscous creep strain (Δε) was obtained by extrapolating the slope of the best-fit line of the data between t = 500 s and t = 600 s for each respective creep curve back to t = 0 s and subtracting this y-intercept from the final strain value at t = 600 s.

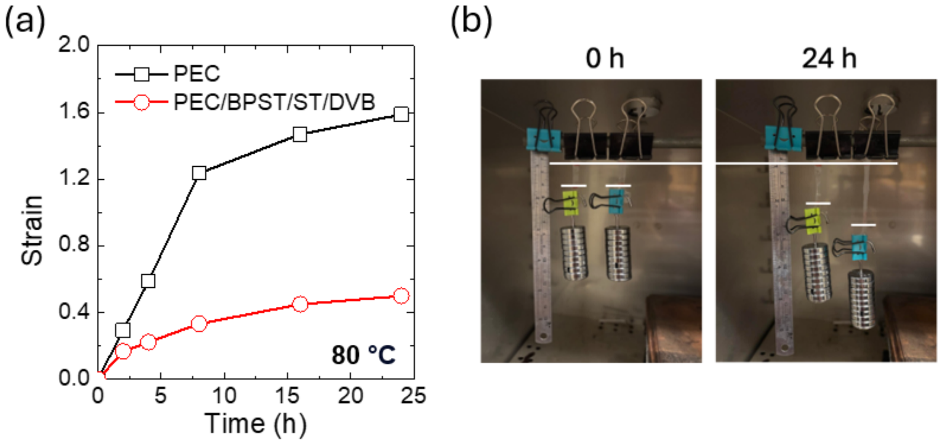

Figure S10. (a) Strain as a function of time for neat PEC and the 1st mold of PEC/BPST/ST/DVB at 80 °C under a tensile load of 0.65 MPa. (b) Image of neat PEC (right) and PEC/BPST/ST/DVB (left) before and after tension mode creep test at 80 °C under a tensile load of 0.65 MPa for 24 h.

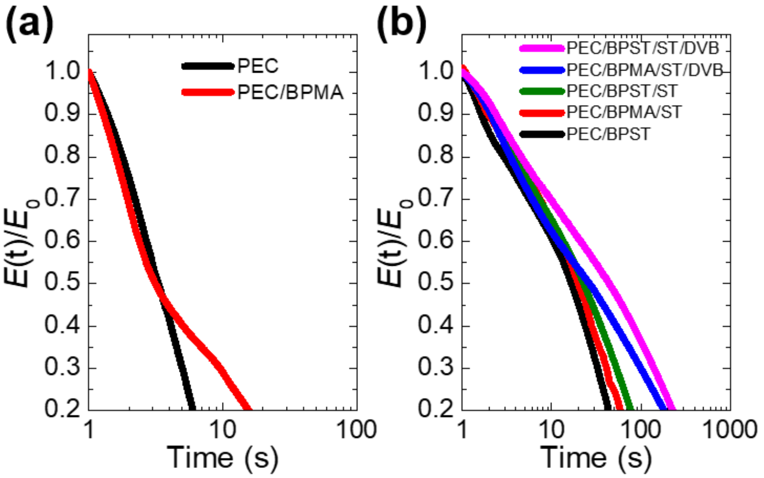

Figure S11. Normalized stress relaxation curves of (a) neat PEC, PEC/BPMA, and (b) 1st mold PEC CANs at 160 °C. The curves are fitted using the KWW stretched exponential decay function to obtain fitting parameters (τ^*, β) and the calculated average relaxation times ($\langle\tau\rangle$).

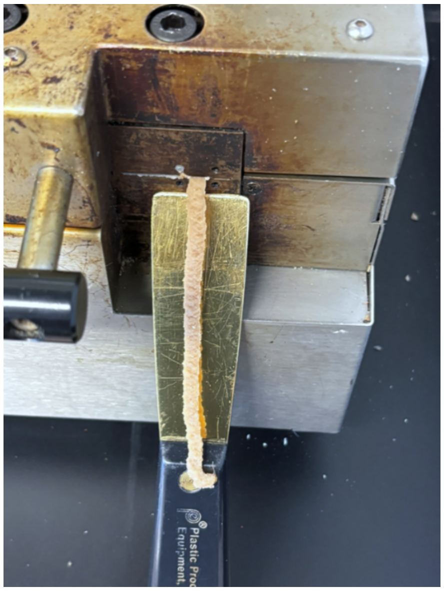

Figure S12. Demonstration of extrudability of PEC/BPST/ST/DVB by feeding 1st mold PEC/BPST/ST/DVB into a twin-screw extruder at 180 °C, resulting in 1st extrudate.

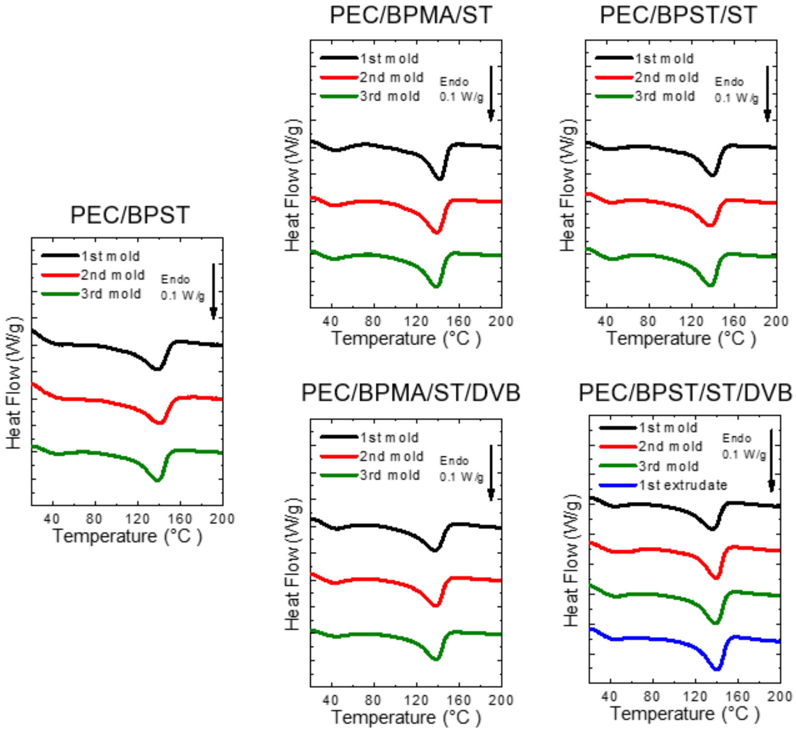

Figure S13. DSC thermograms for 1st, 2nd, and 3rd mold (as well as 1st extrudate for PEC/BPST/ST/DVB) PEC CANs, indicating complete property recovery.

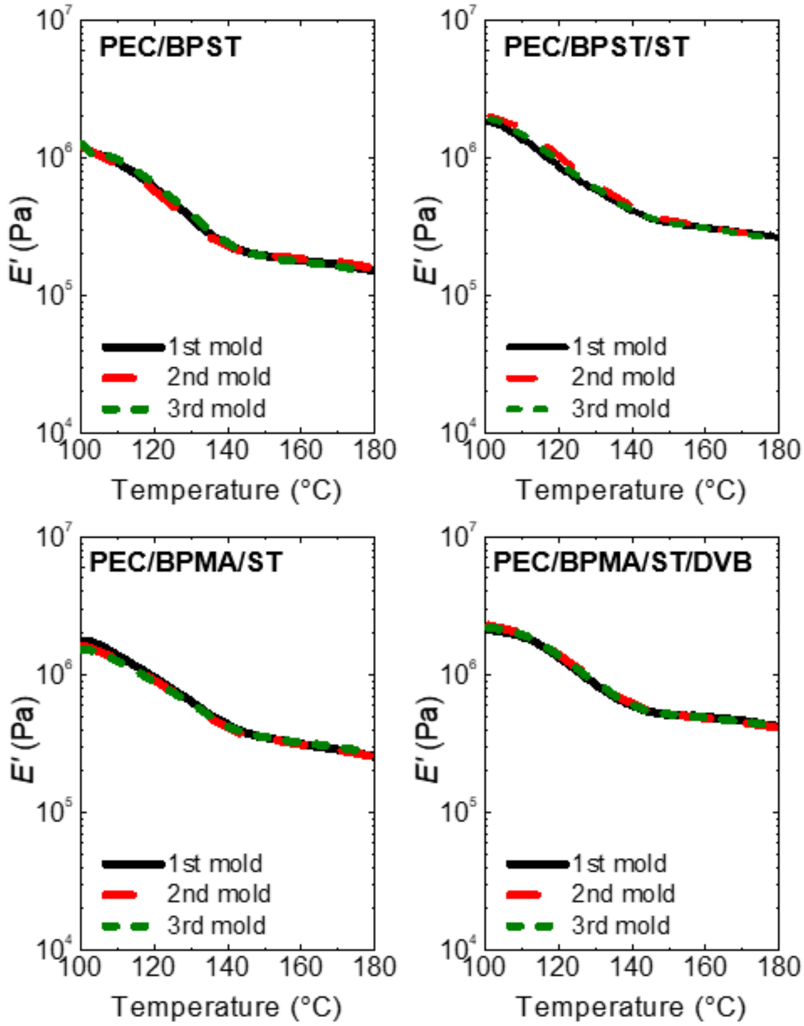

Figure S14. Tensile storage modulus (E') as a function of temperature for 1st, 2nd, and 3rd mold PEC CANs, exhibiting full recovery of cross-link density within experimental error after two reprocessing cycles.

Table S1. Maximum possible cross-link density and experimentally determined effective cross-link density (at 160 °C) of PEC CANs

Sample	Maximum Possible Cross-link Density (mol m^{-3})[a]	Effective Cross-link Density (mol m^{-3}) @160 °C[b]
**PEC/BPST**	86	15 ± 3
**PEC/BPMA/ST**		29 ± 3
**PEC/BPST/ST**		30 ± 3
**PEC/BPMA/ST/DVB**	129	45 ± 4
**PEC/BPST/ST/DVB**		48 ± 2

[a] The maximum possible cross-link density was calculated based on the number of moles of cross-linker per unit volume of PEC.
[b] The effective cross-link density in the rubbery plateau was calculated using the equation $\nu = E / (3RT)$ based on Flory's ideal rubber elasticity theory, with E' used to approximate $E(T)$.[S3]

Table S2. Power-law exponents (n)[a] from creep compliance-time log–log plots at 500–600 s at 160 °C.

Sample	n	R^2
**PEC**	0.98	0.999
**PEC/BPMA**	0.89	0.998
**PEC/BPST**	0.49	0.999
**PEC/BPMA/ST**	0.42	0.997
***PEC/BPST/ST**	0.37	0.991
**PEC/BPMA/ST/DVB**	0.15	0.907
**PEC/BPST/ST/DVB**	0.18	0.930

[a] with $\Delta\varepsilon \sim (\Delta t)^n$

Table S3. Thermal and thermomechanical properties of PEC CANs as a function of the molding steps

Sample	Mold	$T_{m,peak}$ (°C)[a]	$T_{m,endpoint}$ (°C)[a]	χ_c[b]	*E'* at 160 °C[c]
PEC/BPST	1st mold	138	152	3%	0.16 ± 0.03
	2nd mold	138	153	3%	0.15 ± 0.04
	3rd mold	139	152	4%	0.15 ± 0.02
PEC/BPMA/ST	1st mold	140	151	3%	0.31 ± 0.03
	2nd mold	139	151	3%	0.29 ± 0.04
	3rd mold	139	152	3%	0.28 ± 0.03
PEC/BPST/ST	1st mold	139	151	3%	0.32 ± 0.03
	2nd mold	138	151	3%	0.33 ± 0.03
	3rd mold	138	150	3%	0.34 ± 0.03
PEC/BPMA/ST/DVB	1st mold	137	150	2%	0.49 ± 0.04
	2nd mold	137	150	2%	0.45 ± 0.03
	3rd mold	137	149	2%	0.46 ± 0.05
PEC/BPST/ST/DVB	1st mold	136	149	2%	0.52 ± 0.02
	2nd mold	138	149	3%	0.50 ± 0.03
	3rd mold	138	149	3%	0.50 ± 0.05
	1st extrudate	138	149	3%	0.45 ± 0.05

[a]Determined from DSC measurements, listed values are ± 1 °C
[b]Crystallinity, calculated as the ratio of the latent heat of fusion from propylene units measured from DSC to the latent heat of fusion for 100% crystalline PP (207.1 J/g),[S4] listed values are ± 1 %
[c]Determined by DMA; error bars represent one standard deviation of three measurements

Table S4. Room-temperature tensile properties of PEC CANs as a function of the molding step

Sample	Mold	Young's modulus[a] (MPa)	Tensile strength[a] (MPa)	Strain-at-break[a] (%)
PEC/BPST/ST/DVB	1st mold	16 ± 4	13 ± 2	330 ± 50
	2nd mold	14 ± 4	10 ± 2	330 ± 20
	3rd mold	14 ± 3	10 ± 3	310 ± 40
	1st extrudate	18 ± 3	9 ± 2	280 ± 30

[a]Obtained from tensile testing (strain rate = 0.38 s^{-1})

Reference

[S1] M. J. Suazo, J. M. Torkelson, *ACS Appl. Polym. Mater.* **2024**, *6*, 9209-9218.

[S2] Y.-W. Huang, M. J. Suazo, S. M. Barbon, H. A. Brown, E. Auyeung, C. L. P. Shan, J. M. Torkelson, *ACS Macro Lett.* **2025**, *14*, 341-348.

[S3] Flory, P. J. *Principles of Polymer Chemistry*; Cornell University Press, **1953**.

[S4] H. S. Bu, S. Z. Cheng, B. Wunderlich, *Makromol. Chem., Rapid Commun.* **1988**, *9*, 75-77.
